# Altimetry Method for an Interferometric Radar Altimeter Based on a Phase Quality Evaluation

**DOI:** 10.3390/s23125508

**Published:** 2023-06-12

**Authors:** Jong-Soo Ha, Sung-Yong Hong

**Affiliations:** 1 Agency for Defense Development, Yuseong P.O. Box 35, Daejeon 34186, Republic of Korea; jsha@add.re.kr; 2 Department of Radio and Information Communication Engineering, Chungnam National University, 99 Daehak-ro, Yuseong-gu, Daejeon 34134, Republic of Korea

**Keywords:** interferometric radar altimeter, synthetic aperture radar, delay/Doppler radar altimeter, phase comparison monopulse technique, terrain-referenced navigation, phase quality evaluation

## Abstract

A radar altimeter (RA) is useful to improve autonomous functions such as landing guidance or navigation control of an aircraft. To ensure more precise and safer flights by aircraft, an interferometric RA (IRA) capable of measuring the angle of a target is required. However, the phase-comparison monopulse (PCM) technique used in IRAs has a problem in that an angular ambiguity arises with respect to a target with multiple reflection points, such as terrain. In this paper, we propose an altimetry method for IRAs that reduces the angular ambiguity by evaluating the quality of the phase. The altimetry method as introduced here is sequentially described based on synthetic aperture radar, a delay/Doppler radar altimeter, and PCM techniques. Finally, a phase quality evaluation method is proposed for use in the azimuth estimation process. Aircraft captive flight test results are presented and analyzed, and the validity of the proposed method is examined.

## 1. Introduction

Terrain-referenced navigation (TRN) [1,2,3] is a navigation technology that estimates the current location on a map by comparing the measured altitude with the altitude from a digital elevation model (DEM). TRN has the advantage of being able to operate in the face of hostile interference to global positioning systems (GPS) but has the disadvantage of being vulnerable to altitude errors in the employed altimeter and DEM.

An altimeter is a key sensor that determines the performance of TRN. Depending on the method, barometric altimeters, laser altimeters, and radar altimeters can be used, among others. Radar altimeters [4,5,6,7] have the advantage of being able to operate even in poor weather conditions, unlike laser altimeters [8,9], because they have good permeability to clouds and fog. However, due to the wide beam width of the antenna, not only the altitude of the target directly below the beam but also the altitude of the target at the edge of the beam can be considered as that of the target directly below, resulting in altitude errors.

To minimize these errors, researchers have investigated radar altimeters for aircraft to measure not only the range of the target but also the angle of the target [10,11,12,13,14]. Among these altimeters, an interferometric radar altimeter (IRA) [10,11,12] estimates the target angle using the phase comparison monopulse (PCM) technique [15,16] with monopulse radar [17,18]. Unlike conventional radar altimeters that provide only one-dimensional range information, an IRA can provide three-dimensional information (range/elevation/azimuth) about the target and reduce altitude errors.

However, the PCM technique, which estimates the target angle by comparing the phase differences of the target signals between the receiving antennas, assumes a single target with no phase error. Therefore, if the phases of multiple target signals are overlapped or if the phase error increases for some reason, ambiguity arises in the angle estimation and the accuracy decreases [19,20,21]. This angular ambiguity often occurs in IRAs because these devices acquire reflected signals from the terrain with multiple reflectors.

In this paper, we propose an altimetry method that evaluates the phase quality of target signals and estimates the most reliable angle with the lowest level of error. In order to explain the proposed method, the process of deriving three-dimensional information (range/elevation/azimuth) of the target is described in Section 2. In Section 2.1, an altimetry concept for IRAs is introduced. In Section 2.2, the design of the transmitted signal to measure the relative range to the terrain based on radar theory is described. In order to derive the angle (=elevation) of the target, zero Doppler filtering [22] suitable for real-time processing on an aircraft is utilized, as described in Section 2.3. 

In Section 2.4, before undertaking range fast Fourier transform (FFT), range cell migration compensation (RCMC) processing in the time domain is presented based on the synthetic aperture radar (SAR) theory [23]. In Section 2.5, an angle (=azimuth) estimation technique based on multiple-baseline interferometry is introduced. In Section 2.6, the limitations of the conventional angle estimation technique are analyzed. In order to overcome these limitations, the method for estimating the most reliable angle by means of a phase quality evaluation is proposed in Section 2.7. In Section 2.8, the validity of the proposed method is verified by analyzing actual aircraft test data. Finally, we conclude the paper in Section 3.

## 2. Proposed Method for IRAs

### 2.1. Altimetry Concept of IRAs

**Figure 1:**
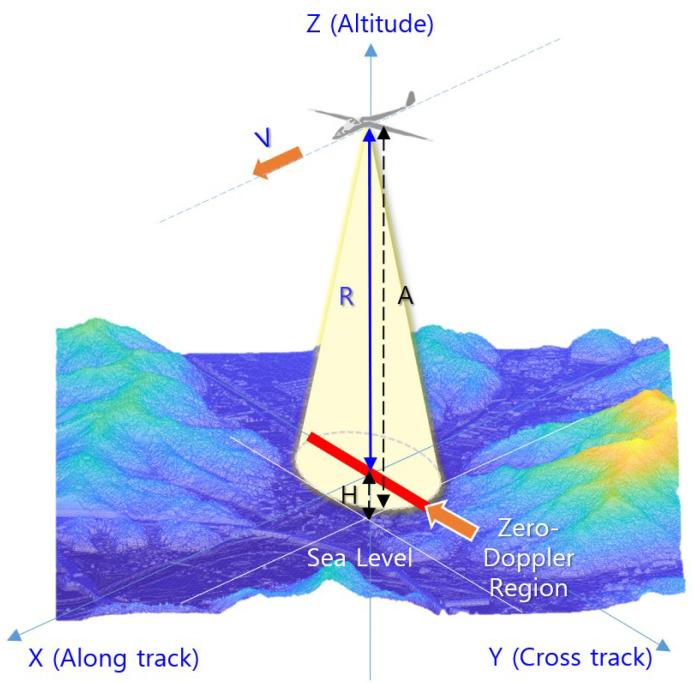
Operational concept of TRN using an IRA.

The operational concept of TRN with an IRA is shown in Figure 1. In this figure, TRN on a cruising aircraft uses an IRA to measure the vertical range (D) to the zero Doppler region within the antenna beam width. It then navigates by deriving the height of the terrain (H(=A−D)) using the altitude from sea level (A) and by estimating its current position by comparing H to the height of the DEM [24,25].

**Figure 2:**
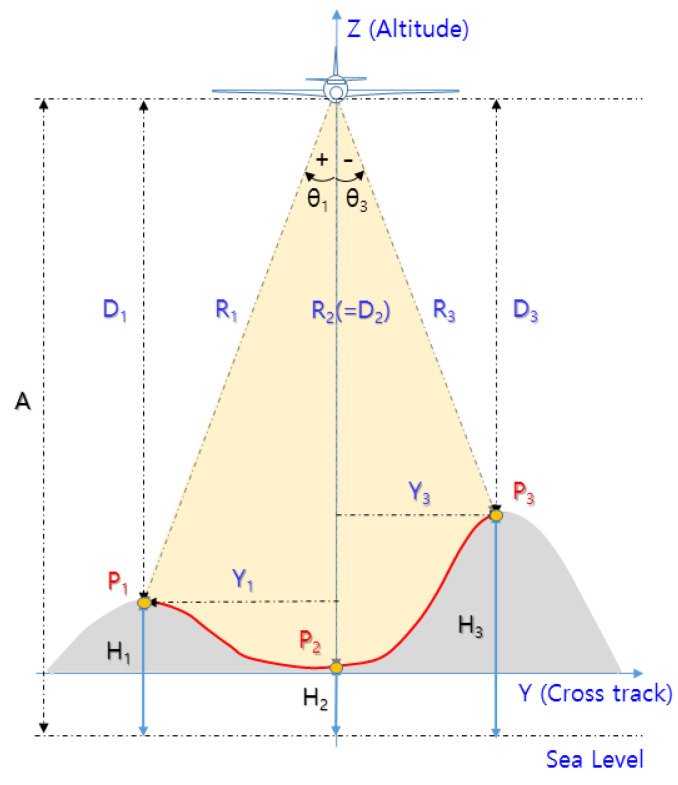
Geometry of altitude error in terrain.

However, as shown in Figure 2, the H of the terrain in the zero Doppler region ($P_{1}$∼$P_{3}$) varies as $H_{2}$∼$H_{3}$ depending on the shape of the terrain. Accordingly, if the angle of the reflection point is not known, measurement error of the target range (R) occurs. For example, if the shortest range ($R_{3}$) at point $P_{3}$ is regarded as the vertical range ($D_{2}$), the measurement error is $\left|R_{2}-R_{3}\right|$.

Given that the IRA can estimate the target angle (=azimuth) based on the PCM technique [15], estimating the angle (θ) with target range (R) can minimize the measurement error, as shown in Figure 2. For example, at point $P_{3}$, the vertical range ($D_{3}$) and the horizontal range ($Y_{3}$) can be calculated based on $R_{3}$ and $\theta_{3}$, meaning that TRN can navigate by comparing the height of the DEM with $H_{3}$ at point $P_{3}$.

### 2.2. Design of the Transmitted Signal for Computation of the Target Range

The IRA described in this paper transmits and receives linear frequency modulation (LFM) pulses to measure the altitude of distant terrain, as shown in Figure 1. The transmitted signal ($S_{t}$) can be defined using Equation (1).

(1) $$S_{t}=A_{t}\exp\left(j2\pi \left(f_{c}\left(t\right)+\frac{k_{e}}{2}t^{2}\right)\right)$$

In Equation (1), $A_{t}$ is the magnitude of the transmitted signal, $f_{c}$ is the carrier frequency, and t is the time. $k_{e}(=B/T_{p})$ is the slope of the frequency modulation, which varies depending on the frequency bandwidth (B) and the pulse width ($T_{p}$). $T_{p}$ should be designed to be small to reduce the blind zone of the transmission time. However, because $T_{p}(=1/B_{p})$ is proportional to the signal-to-noise ratio (*SNR*) of the received signal, as shown in Equation (2) [26], it is necessary to analyze the *SNR* before designing the transmitted signal.

(2) $$SNR=\frac{P_{t}\cdot G_{t}\cdot G_{r}\cdot \lambda^{2}\cdot \sigma \cdot N_{PRF}}{{\left(4\pi \right)}^{3}\cdot R^{4}\cdot KT_{0}\cdot F\cdot B_{p}\cdot L_{T}}$$

In Equation (2), $P_{t}$ is the transmission power, $G_{t}$ is the transmission antenna gain, $G_{r}$ is the receiving antenna gain, λ is the wavelength, σ is the target radar cross section (RCS), $N_{PRF}$ is the pulse repetition frequency (*PRF*) number in one coherent processing interval (CPI), R is the range to the target, K is the Boltzmann constant, $T_{0}$ is the absolute temperature, F is the noise figure (NF), and $L_{T}$ is the total loss including the system loss and the propagation loss.

**Figure 3:**
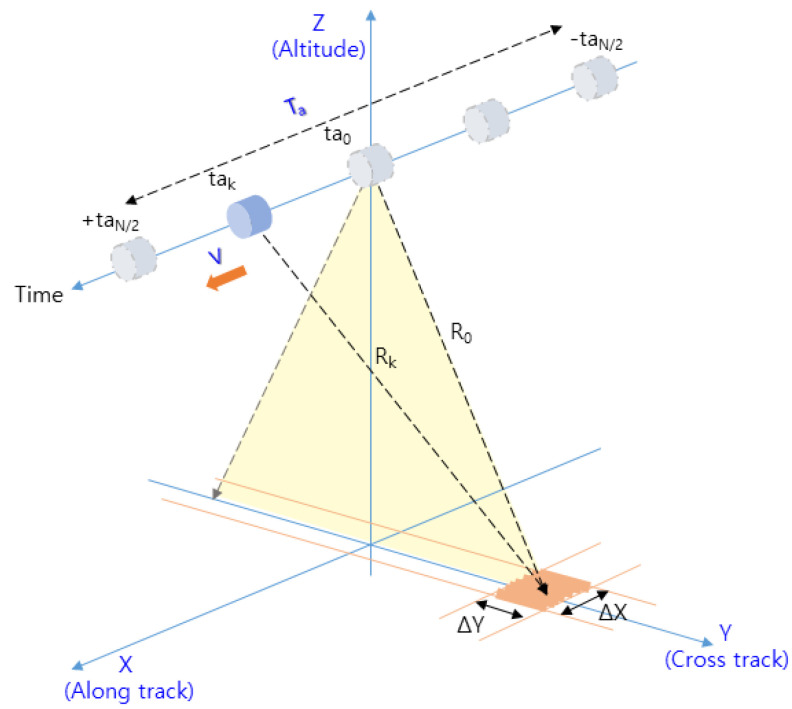
Geometry of the IRA in the flight path.

Figure 3 shows the geometry of the IRA’s reflected signal in the flight path of the cruising aircraft.

In Figure 3, $T_{a}(=t_{a_{N/2}}-(-t_{a_{N/2}}))$ is the aperture time [23], which represents the time during one CPI, as expressed in Equation (3). The pulse repetition interval (*PRI*) is equal to 1/*PRF*, L is the length traveled during $T_{a}$, V is the velocity of the aircraft, and ΔX is the resolution of the along track.

(3) $$T_{a}=\frac{L}{V}=\frac{R\lambda }{2V\cdot ∆X}=PRI\cdot N_{PRF}$$

If the resolution of the cross track is ΔY and the inherent reflection coefficient of an object with an arbitrary material is $\sigma_{0}$, σ can be represented by $\sigma_{0}\cdot ∆X\cdot ∆Y$. Therefore, the *SNR* of the terrain signal in Equations (2) and (3) can be expressed as Equation (4). Considering the range resolution (ΔR(=c/2B)) and the angle of the terrain slope (β), ΔY can be simplified to ΔR/sinβ or it can be set to an arbitrary representative value.

(4) $$SNR=\frac{P_{t}\cdot G_{t}\cdot G_{r}\cdot \lambda^{2}\cdot \sigma \cdot ∆Y\cdot PRF}{{\left(4\pi R\right)}^{3}\cdot KT_{0}\cdot F\cdot B_{p}\cdot L_{T}\cdot 2V}$$

By calculating a $T_{p}$ that satisfies the minimum *SNR* for detecting the terrain in Equation (4), the LFM pulse can be designed by combining B and *PRF*. However, considering the sampling frequency ($f_{s}$) that satisfies the Nyquist sampling theorem, the altitude bin (ΔH) that can be processed without aliasing, i.e., without range ambiguity, can be obtained as shown in Equation (5). Thus, it is necessary to consider these parameters in the design stage of the transmitted signal.

(5) $$\Delta H=\frac{T_{p}}{B}\cdot \frac{f_{s}}{2}\cdot \frac{c}{2}$$

### 2.3. Employing Conventional Zero Doppler Filtering to Derive the Target Elevation

Regarding the acquisition of the three-dimensional information of the target, the target range computation described earlier is important. In addition to the acquisition of the azimuth, which will be described later, it is necessary to acquire the angle (=elevation; in the three-dimensional (3D) radar, the angle is categorized as azimuth and elevation; since the elevation is used in the DEM to indicate the height, it is expressed as the angle (=elevation) for clarity) of the flight direction. In this paper, instead of acquiring the elevation of the target, the zero Doppler filtering of the delay/Doppler radar altimeter (DDA) technique [22] is employed to derive the elevation region, and the RCMC is processed in the time domain.

**Figure 4:**
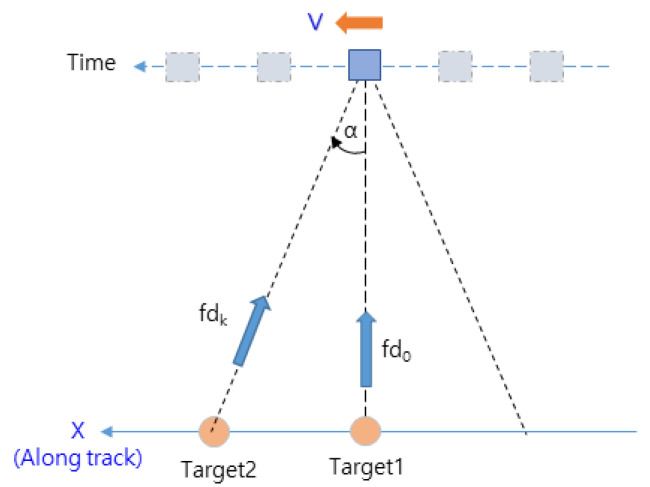
Doppler frequency of the target for the flight path.

Zero Doppler filtering in the DDA technique is a part of the range Doppler algorithm of the SAR technique [23]. It is performed only until the azimuth FFT; then, only the frequency signals in the zero Doppler region shown in Figure 1 are acquired. Compared to the range Doppler algorithm, it is less computationally intensive, which has the advantage of enabling real-time processing in aircraft flying at high speeds. In this regard, the concept of the target’s Doppler frequencies for the flight path is shown in Figure 4.

The Doppler frequency acquired by an aircraft flying at a constant altitude, as shown in Figure 4, is determined by Equation (6) [23]. Because $f_{d}$ is zero in the vertical direction of the aircraft, instead of acquiring the entire terrain signal, zero Doppler filtering acquires only the terrain signal at the zero Doppler point, such as $f_{d0}(\alpha =0)$, excluding the components $f_{dk}(\alpha \neq 0)$. In this way, the elevation region to be computed can be derived.

(6) $$f_{d}=\frac{2V}{\lambda }\cdot \sin\alpha$$

**Figure 5:**
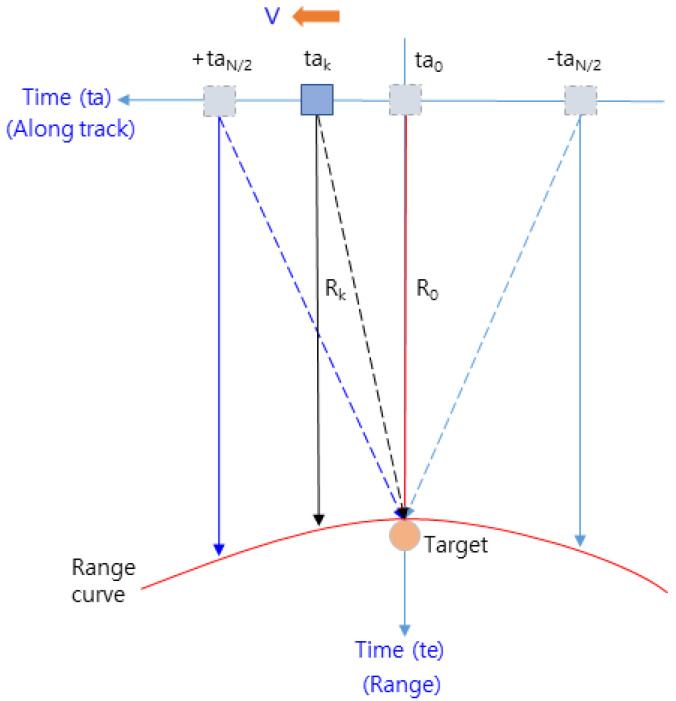
Range curve of the target for the flight path.

In order to perform zero Doppler filtering using the DDA technique, the azimuth FFT of the range Doppler algorithm should be processed beforehand. Therefore, it is necessary to compile a number of data in the along-track direction, which correspond to the amount of data collected during $T_{a}$ in Figure 3. If we plot the detected range ($R_{k}$) for one target at each observation point ($t_{a_{k}}$) parallel to the range (=time $t_{e}$) axis while moving along the track, we can plot a range curve approximated by the red line shown in Figure 5. This is the range cell migration (RCM) process of SAR processing, requiring RCMC processing to compensate for it.

The received signal ($S_{r}$) for the transmitted signal in Equation (1) is converted to a baseband signal and is determined by Equation (7). The conventional range Doppler algorithm performed the azimuth FFT on this signal and compensated for the range curve in Figure 5 through RCMC processing [23].

(7) $$S_{r}\left(t\right)=A_{r}\exp\left(j2\pi \left(-f_{c}\tau \left(t\right)+\frac{k_{e}}{2}{\left(t-\tau \left(t\right)\right)}^{2}\right)\right)=A_{r}\exp\left(j2\pi \left(-f_{c}\tau_{0}+\frac{k_{e}}{2}t^{2}-k_{e}\tau_{0}t+\frac{k_{e}}{2}\tau_{0}^{2}\right)\right)$$

In Equation (7), $A_{r}$ is the magnitude of the received signal, and τ(t) is the delay time of the reflected signal from the target, which can be expressed as Equation (8). In Equation (8), compared to $f_{c}$, both $f_{d}$ and t are negligibly small. Accordingly, in this paper, we assume that $\tau (t)≅\tau_{0}$.

(8) $$\tau \left(t\right)=\frac{2R\left(t\right)}{c}=\frac{2R_{k}}{c}-\frac{2V\cdot \sin\alpha }{c}\cdot t=\frac{2R_{k}}{c}-\frac{f_{d}}{f_{c}}\cdot t≅\frac{2R_{k}}{c}=\tau_{0}$$

### 2.4. RCMC Processing in the Time Domain for Zero Doppler Filtering

The IRA undertakes deramping before converting the received signal ($S_{r}$) to the baseband signal for real-time processing. Thus, $S_{r}$ can be calculated according to Equation (9) instead of Equation (7). In Equation (9), it is difficult to employ conventional RCMC processing as is.

(9) $$S_{r}\left(t\right)=A_{r}\exp\left(j2\pi \left(f_{c}\tau_{0}+k_{e}\tau_{0}t+\frac{k_{e}}{2}\tau_{0}^{2}\right)\right)$$

In order to overcome this difficulty, in this paper, we present a method to compensate for the range curve by performing RCMC processing in the time domain before the azimuth FFT, although it is not as accurate as conventional RCMC processing performed in the frequency domain after the azimuth FFT.

The target range ($R_{k}$) detected at each observation point ($t_{a_{k}}$) in Figure 5 can be expressed as Equation (10) [23], where $R_{0}$ is the closest range in Figure 5 and $∆R_{k}$ is the RCM component that forms the range curve indicated by the red line in Figure 5.

(10) $$R_{k}=\sqrt{R_{0}^{2}+V^{2}{\left(t_{a_{k}}-t_{a_{0}}\right)}^{2}}≅R_{0}+∆R_{k}=R_{0}+\frac{V^{2}{\left(t_{a_{k}}-t_{a_{0}}\right)}^{2}}{2R_{0}}$$

Decomposing Equation (9) into the azimuth time ($t_{a}$) and the range time ($t_{e}$) in Figure 5 and substituting ${2R}_{k}/c$ of Equation (8) into $\tau_{0}$ of Equation (9), Equation (9) can be written as Equation (11).

(11) $$S_{r}\left(t_{a},t_{e}\right)=A_{r}\exp\left(j\frac{4\pi R_{k}}{\lambda }+j\frac{4\pi k_{e}}{c}R_{k}t_{e}-j\frac{4\pi k_{e}}{c^{2}}R_{k}^{2}\right)$$

Performing the range FFT operation on Equation (11) transforms it into Equation (12).

(12) $$S_{r}\left(t_{a},t_{e}\right)=A_{r}\mathrm{sinc}\left(f_{e}-k_{e}\frac{2R_{k}}{c}\right)\cdot \exp\left(j\frac{4\pi R_{k}}{\lambda }-j\frac{4\pi k_{e}}{c^{2}}R_{k}^{2}\right)$$

In Equation (12), $f_{e}$ denotes the range frequency. The range (=time $t_{e}$) axis of the received signal was transformed into the frequency domain, and the center of the sinc function is $k_{e}\frac{2R_{k}}{c}$.

**Figure 6:**
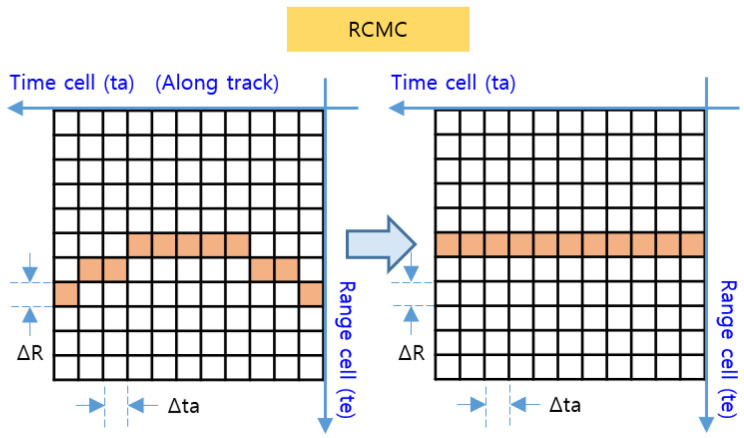
RCMC processing in the time domain.

Given that $R_{k}$ varies at every point ($t_{a_{k}}$) in Equation (10), ${∆R}_{k}$ is added to the closest range ($R_{0}$) at every point ($t_{a_{k}}$) in Equation (12). Consequently, in this paper, we calculate ${∆R}_{k}$ for each observation point ($t_{a_{k}}$) and move the range cell by an amount equal to the calculated value, as shown in Figure 6. However, the proposed method has the advantage of real-time processing, but the accuracy is somewhat decreased because errors arise depending on the accuracy of $R_{0}$ and V and due to the error based on the size of ∆R.

**Figure 7:**
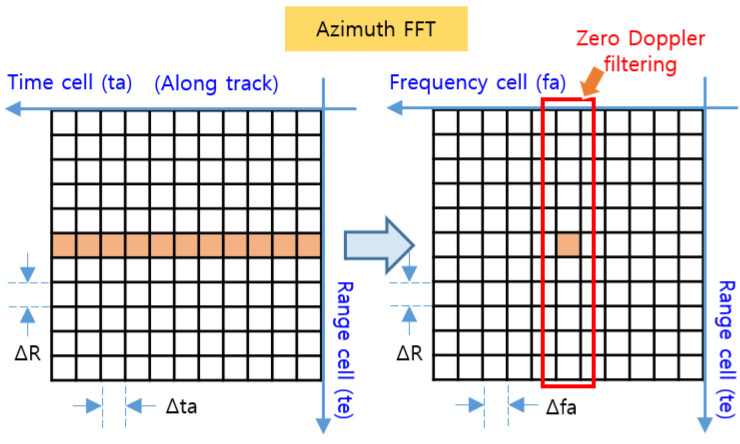
Azimuth FFT and zero Doppler filtering.

In Figure 6, the target signal aligned to the range cell by RCMC processing is the signal on the range axis and the time axis, where the range axis corresponds to the range time ($t_{e}$) and the time axis corresponds to the azimuth time ($t_{a}$) in Figure 5. By performing the azimuth FFT on this time axis, the target signal with target range $\tau_{tgt}$ and Doppler frequency $f_{d_{tgt}}$ can be obtained, as shown in Figure 7. The time axis is then converted to the frequency axis, and the target’s elevation region can be derived toward the direct downward direction by means of zero Doppler filtering, which detects only signals with a zero Doppler frequency in order to extract only signals for which $f_{d_{tgt}}=0$.

### 2.5. Conventional Angle-Estimation Technique Based on Multiple Baseline Interferometry

The computation of the target’s range and elevation to acquire three-dimensional information of the target was described earlier. In this section, we describe the estimation of the target’s azimuth (orthogonal to the direction of flight). The target angle estimation is based on the PCM technique of monopulse radar. However, considering that it is difficult to resolve the angular ambiguity with a typical single-baseline antenna, in this paper, we employ a method based on a multiple-baseline antenna to estimate the azimuth of the target by comparing the phase differences of the target signals received by each antenna, as in several earlier studies [14,27].

**Figure 8:**
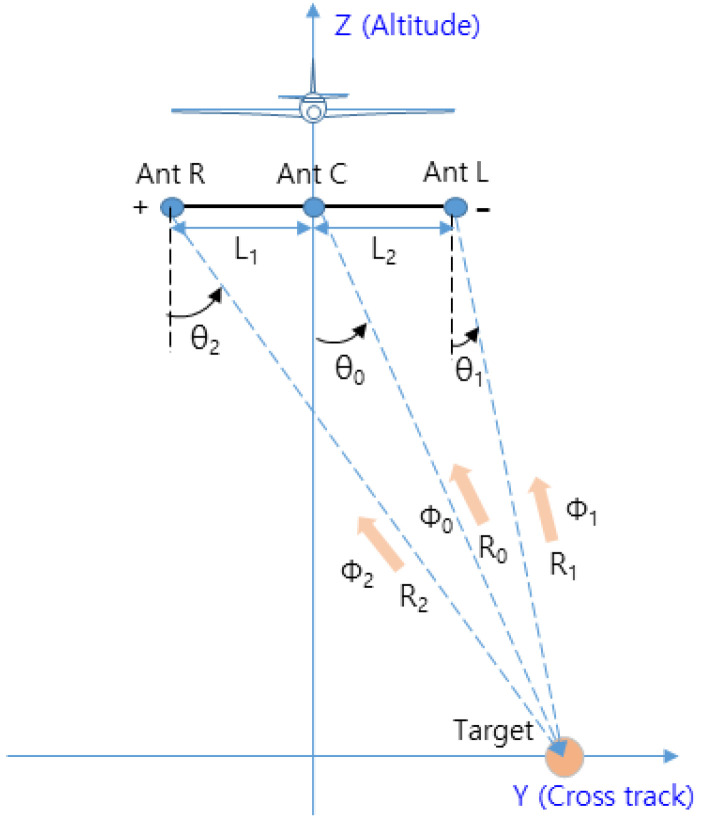
Geometry of multiple-baseline interferometry.

In Figure 8, Ant L (Left), Ant C (Center), and Ant R (Right) are placed with interantenna spacings of $L_{1}$ and $L_{2}$, respectively, and the reflected signals from the target within the zero Doppler region are received by each antenna. Equation (13) can be derived from the geometry in Figure 8 for range $R_{k}$ and phase $\emptyset_{k}$ of the received signal.

(13) $$\sin\left(\theta_{k}\right)=-\frac{\lambda }{L_{k}}\left(\frac{∆\emptyset_{k}}{2\pi }+m_{k}\right)+\frac{L_{k}}{2R_{k}},(k=1,2)$$

In Equation (13), $L_{k}$ is the interantenna spacing, and $\theta_{k}$ is the angle of the target obtained from Ant L and Ant R. $m_{k}$ is a variable representing the periodicity of the measured phase difference ${∆\emptyset }_{k}(=\emptyset_{k}-\emptyset_{0})$, which has angular ambiguity that occurs at every period (2π) [28].

To resolve this angular ambiguity, the assumption that $\theta_{0}$ is most reliable when the difference (${∆\theta }_{min}$) between $sin\left(\theta_{1}\right)$ and $sin\left(\theta_{2}\right)$ is minimized is used to obtain $m_{k}$, as shown in Equation (14). The obtained values of $sin\left(\theta_{1}\right)$ and $sin\left(\theta_{2}\right)$ are arithmetically averaged, as shown in Equation (15), to obtain the estimated angle ($\hat{\theta }$) of $\theta_{0}$ [18,27]. From the range ($R_{0}$) and angle ($\hat{\theta }$) obtained in this way, the vertical range ($D_{k}(=R_{0}\cos\hat{\theta })$) in the direct downward direction can be estimated.

(14) $${∆\theta }_{min}={min}_{m_{1},m_{2}}\left|\sin{(\theta }_{1})-sin(\theta_{2})\right|,\left|\theta_{1,2}\right|\leq \theta_{width}$$

(15) $$\hat{\theta }=\mathrm{asin}\left(\frac{sin(\theta_{1})+sin(\theta_{2})}{2}\right)$$

**Figure 9:**
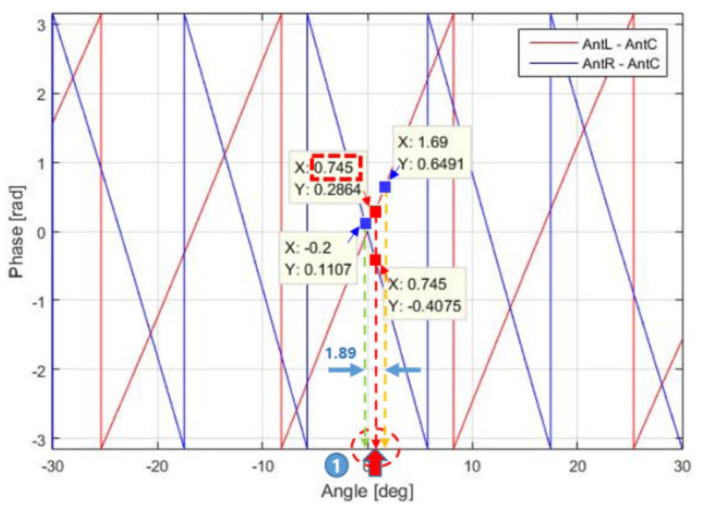
Concept of angle estimation based on the phase difference.

For the multiple-baseline antenna designed in this paper, the values of $\theta_{k}(=\mathrm{asin}\left(\sin\left(\theta_{k}\right)\right))$ for ${∆\emptyset }_{k}$ of Equation (13) is plotted in Figure 9. In Figure 9, the blue line represents the graph of ${∆\emptyset }_{1}$(= the phase difference between Ant R and C) versus $\theta_{1}$ (when k=1), and the red line represents the graph of ${∆\emptyset }_{2}$ (=the phase difference between Ant L and Ant C) versus $\theta_{2}$ (when k=2).

If ${∆\emptyset }_{k}$, as indicated by the red point, is −0.4075 (when k=1) and 0.2864 (when k=2), then $\theta_{1}$ and $\theta_{2}$ are both 0.745°, resulting in ${∆\theta }_{min}$ = 0 and $\hat{\theta }$ = 0.745°, respectively. If ${∆\emptyset }_{k}$, as indicated by the blue point, is 0.1107 (when k=1) and 0.6491 (when k=2), then $\theta_{1}$ = −0.2° and $\theta_{2}$ = 1.69°, resulting in ${∆\theta }_{min}$ = 1.89° and $\hat{\theta }$ = 0.745°, respectively. Therefore, even with the added error in ${∆\emptyset }_{k}$, $\hat{\theta }$ corresponding to Equation (14) can be derived according to Equation (15).

### 2.6. Analysis of the Limitations of the Conventional Angle Estimation Technique

**Figure 10:**
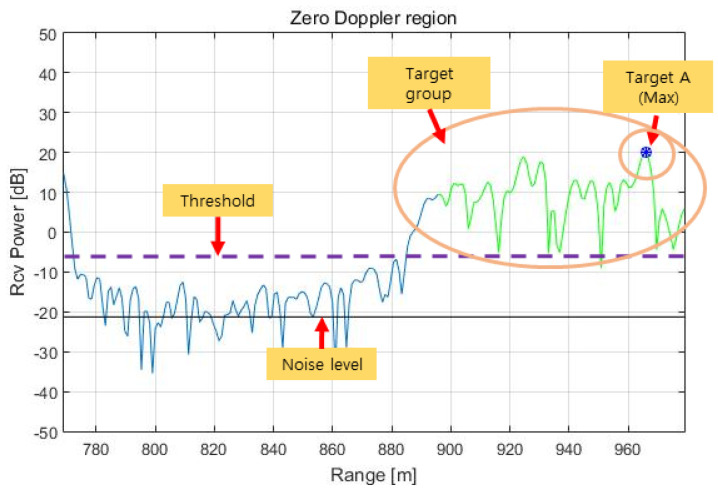
Range spectrum in the zero Doppler region.

Before estimating the azimuth of the target, the peak point of the range cells within the zero Doppler filtering in Figure 7 is specified as target A with zero elevation, as shown in Figure 10. The azimuth is then estimated according to Equations (13)–(15) from the phase information of the target. In this regard, we analyzed and concluded that the conventional angle estimation technique has limitations in two aspects: the target identification process and the angle (=azimuth) estimation process.

First, regarding the target identification process, unlike monopulse radar, which detects a single target, an IRA receives target signals from multiple reflective points on the terrain, meaning that the peak in the range spectrum may be the signal reflected from a single target with a large RCS but may also be superimposed signals from multiple targets with similar ranges and different angles. Therefore, for a single target signal, the phase error may be small, and the estimated angle may be accurate, but for multiple target signals, the phase error increases, resulting in a large angle error.

**Figure 11:**
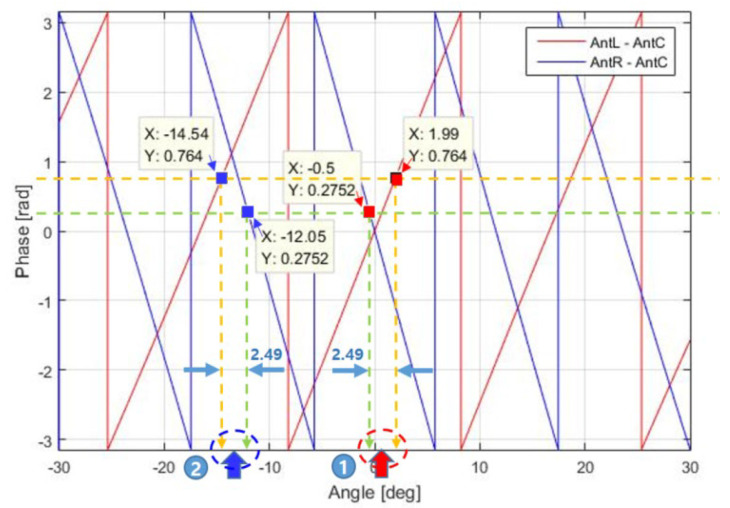
Two targets with the same angle difference.

Second, regarding the angle estimation process, it appears to be reasonable to derive Equations (14) and (15) based on the assumption that $\hat{\theta }$ is most reliable when the difference between $sin\left(\theta_{1}\right)$ and $sin\left(\theta_{2}\right)$ is minimized for a multiple-baseline antenna, but it is difficult to determine the extent to which the result of Equation (15) can be trusted. In this regard, Figure 11 is instructive.

In Figure 11, when ${∆\emptyset }_{1}$ is 0.2752 and ${∆\emptyset }_{2}$ is 0.764, ${∆\theta }_{min}$ is 2.49° in regions 1 and 2, yielding $\hat{\theta }$ = 0.745° in region 1 and $\hat{\theta }$ = −13.295° in region 2. In this case, the angle cannot be estimated using the conventional method because there are two solutions. These two limitations motivated us to consider the phase quality evaluation in this paper.

### 2.7. Azimuth Estimation Based on a Phase Quality Evaluation

In order to overcome the two limitations of the conventional angle estimation techniques described above, we propose a method that evaluates the phase quality of the target signals in the range spectrum, as shown in Figure 10, before estimating the target angle (=azimuth), after which it selects the target whose azimuth is estimated to be the most reliable. In other words, instead of “calculate signal magnitude → select target → estimate azimuth”, the azimuth of the proposed method is estimated in the order of “calculate signal magnitude → select target group → calculate phase quality and estimate azimuth in advance → evaluate phase quality → select the most reliable target → finally estimate azimuth”.

This method takes into account the contamination of the phase information. Just as Equations (14) and (15) in Section 2.6 are derived from the assumption that $\hat{\theta }$ of the conventional multiple-baseline antenna is most reliable when ${∆\theta }_{min}$ is minimized, the proposed method works on the assumption that the better the phase quality is, the more reliable $\hat{\theta }$ is.

First, we set a threshold larger than the noise level, as shown in Figure 10. We classify the signals in a certain region of the range spectrum (e.g., N/2 to N when the total number of samples in the range spectrum is N) into a group of targets. The azimuths of these targets are then estimated according to Equations (14) and (15), and their phase qualities are evaluated as described below.

In this paper, we precalculate and store the phase difference versus the angle (=azimuth) values in Figure 9 because the phase quality could not be determined by the conventional Equations (14) and (15). For example, if the target azimuth is 0.745°, −0.4075 for ${∆\varphi }_{1}$ and 0.2864 for ${∆\varphi }_{2}$ are then calculated and stored in the database (DB) (${∆\varphi }_{k}$ is referred to as the stored value to distinguish it from the measured value (${∆\emptyset }_{k}$)). Then, from the range spectrum in Figure 10 obtained from the aircraft captive flight test (CFT), the target azimuths of the entire target group are calculated. If the target azimuth ($\hat{\theta }$) is determined to be 0.745°, ${∆\emptyset }_{1}$ is equal to 0.1107, and ${∆\emptyset }_{2}$ is equal to 0.6491 in Equation (13), the quality of phase ${∆\emptyset }_{q}$ is then calculated as shown in Equation (16).

(16) $${∆\emptyset }_{q}=\left|{∆\varphi }_{1}-{∆\emptyset }_{1}\right|+\left|{∆\varphi }_{2}-{∆\emptyset }_{2}\right|$$

Subsequently, by comparing the phase quality (${∆\emptyset }_{q}$) of the entire target group, as shown in Equation (17), selecting the mth sample with the minimum value as the target, and estimating the target azimuth ($\hat{\theta }$), the proposed method completes the target azimuth estimation.

(17) $${∆\emptyset }_{q-min}={min}_{m}{∆\emptyset }_{q}\left(m\right),m=m_{1},..,m_{k},..,M$$

**Figure 12:**
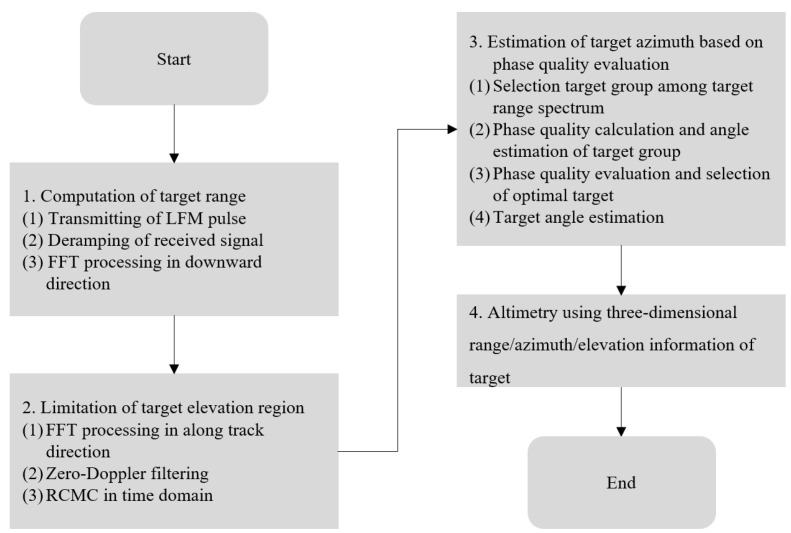
Flow chart of the proposed method.

The proposed method is adopted for use with targets 1 and 2 in Figure 11. For target 1, $\hat{\theta }$ is 0.745°, ${∆\emptyset }_{1}$ is 0.2752, ${∆\emptyset }_{2}$ is 0.764, ${∆\varphi }_{1}$ is −0.4075, and ${∆\varphi }_{2}$ is 0.2864. Thus, according to Equation (16), ${∆\emptyset }_{q}$ is calculated and found to be 1.1603. For target 2, $\hat{\theta }$ is −13.295°, ${∆\emptyset }_{1}$ is 0.2752, ${∆\emptyset }_{2}$ is 0.764, ${∆\varphi }_{1}$ is 0.9424, and ${∆\varphi }_{2}$ is 1.227. Then, ${∆\emptyset }_{q}$ is calculated according to Equation (16) and found to be 1.1302. Therefore, for this sample, −13.295° with the smaller ${∆\emptyset }_{q}(=1.1302)$ is estimated as the target azimuth ($\hat{\theta }$). The subsequent procedure is as described earlier. Figure 12 shows a flow chart of the proposed method.

### 2.8. Aircraft CFT Results

**Figure 13:**
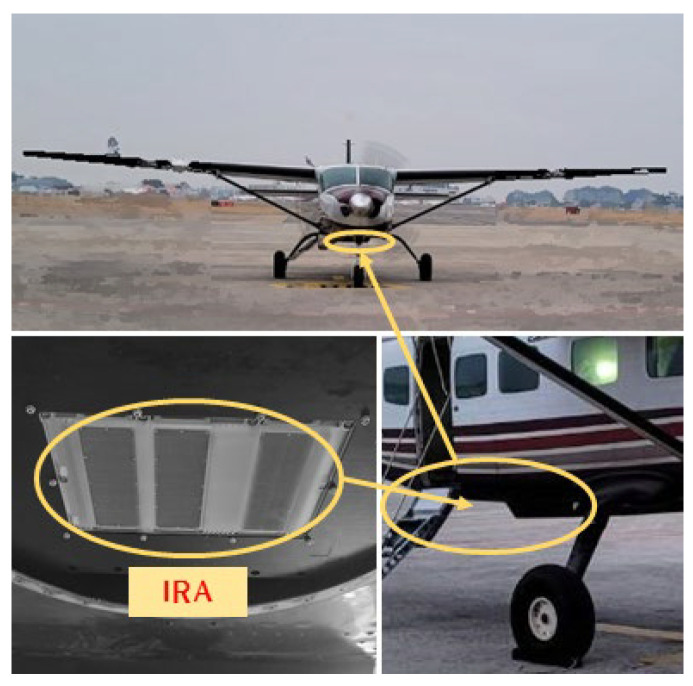
IRA and aircraft.

**Figure 14:**
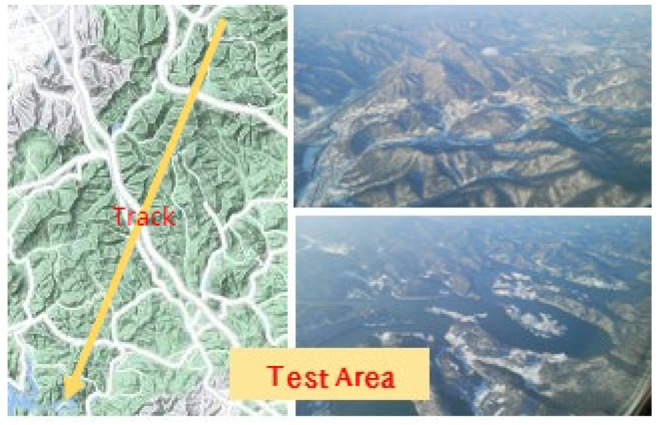
Test area and corresponding path.

The IRA was mounted on the underside of an aircraft, as shown in Figure 13, to conduct an aircraft CFT and acquire test data. The test was conducted in an area near Jeollabuk-do, South Korea, and the plane flew along the route shown in Figure 14, with a flight altitude of about 1.5 km above sea level and a flight speed of about 170 knots.

**Figure 15:**
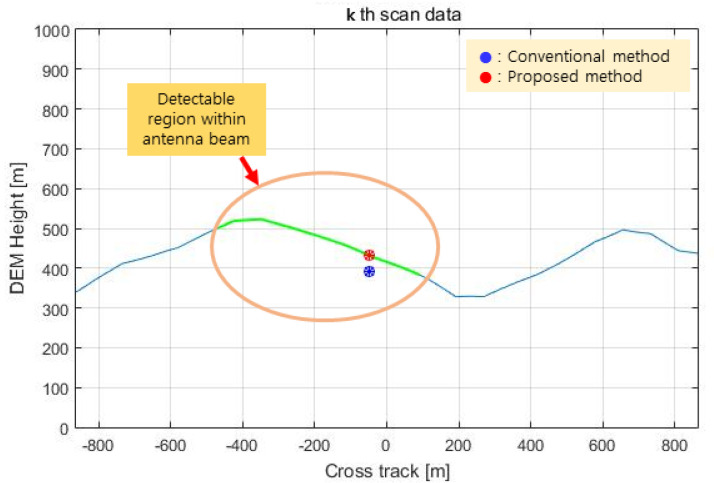
Test result (terrain height in the cross track).

**Figure 16:**
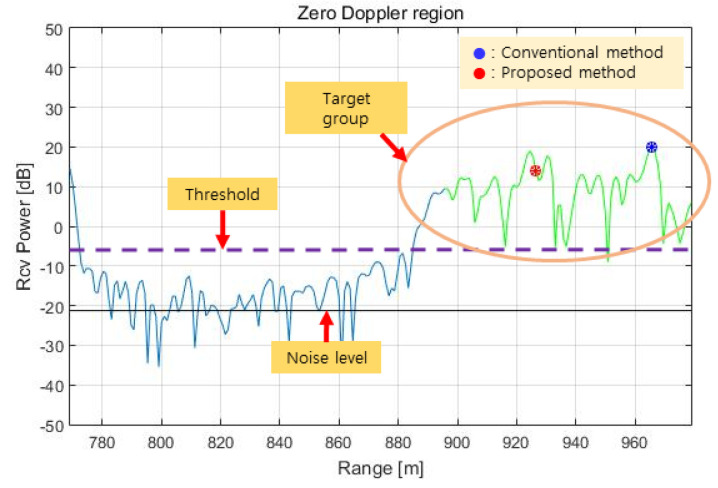
Test result (target range in the zero Doppler region).

**Figure 17:**
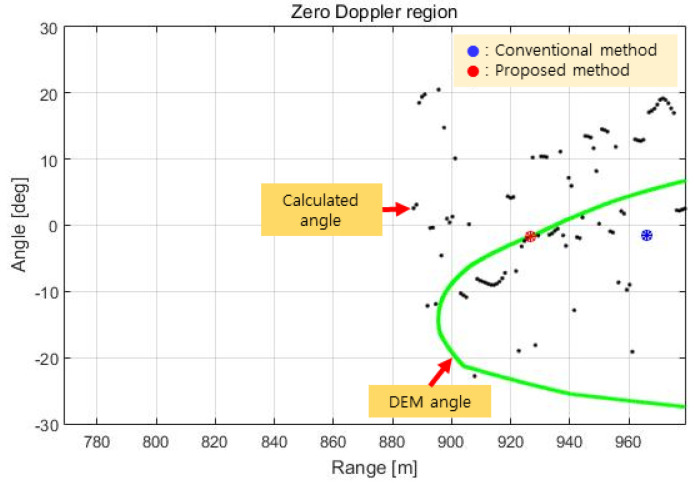
Test result (target azimuth in the zero Doppler region).

The processing results of the kth scan of the flight test data are shown in Figure 15, Figure 16 and Figure 17 below. The height of the DEM in the orthogonal direction of the flight direction is shown in Figure 15. In this figure, the green line indicates the height of the DEM of the detectable region within the antenna beam width of the IRA, the blue dot is the height of the terrain computed by the conventional method, and the red dot is the height of the terrain computed by the proposed method.

The range spectrum in the zero Doppler region obtained by RCMC processing in the time domain, azimuth FFT, and zero Doppler filtering for the above terrain is shown in Figure 16. This range spectrum is identical to the range spectrum in Figure 10, but in Figure 16, we can see the target (red dot) selected as a result of the phase quality evaluation.

The results of the azimuth estimation of the signals above the threshold in Figure 16 are shown in Figure 17. In Figure 17, the green line shows the target angle (=azimuth) on the DEM, and the black dots show the target angles estimated by comparing the phase quality. Figure 17 shows that there is some similarity between the estimated angles and the angles of the DEM, but there are significant angular errors in that the black dots are far off the green line.

This may be an angular ambiguity inherent in the multiple-baseline antenna-based PCM technique [14], an inherent error in the DEM due to the accuracy of DEM production, or a phase error due to the superposition of the phases of multiple targets in one range cell (ΔR).

In this paper, the phase quality containing these errors is compared using Equation (16) to calculate the angle at each point, and the angle ($\hat{\theta }$) is estimated as the target angle by evaluating the phase quality (${∆\emptyset }_{q}$) according to Equation (17) and selecting the mth sample with the minimum value of ${∆\emptyset }_{q}$ as the target. In Figure 17, the red dot represents the target angle estimated by the proposed method, and the blue dot represents the target angle at the maximum signal point according to the conventional method.

## 3. Discussion

**Figure 18:**
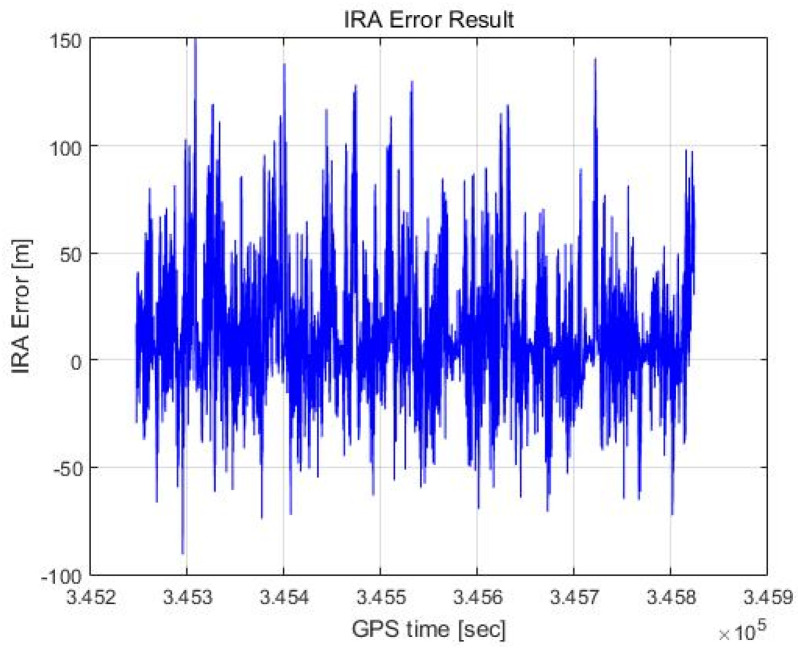
RA error result (max point).

**Figure 19:**
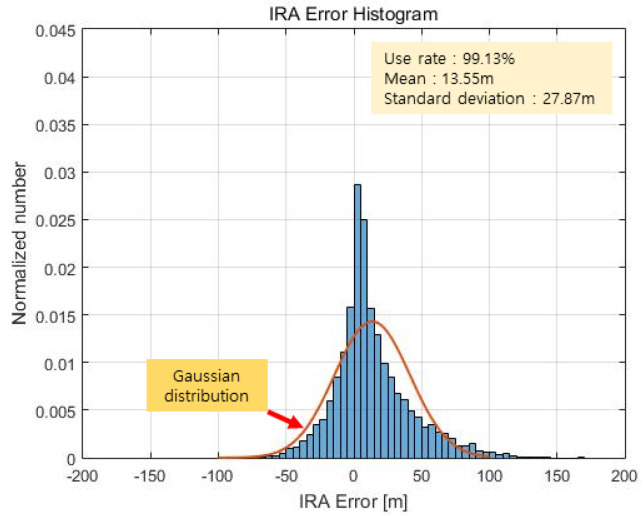
RA error histogram (max point).

In this paper, an altitude calculation for one scan was performed for a total of 11,541 scans with the proposed method, and the error was analyzed by comparing this outcome with the DEM results. Figure 18 and Figure 19 show the altimetry results when RCMC processing in the time domain and zero Doppler filtering as described in Section 2.4 are performed, with the azimuth of the maximum signal point assumed to be 0 degrees without the angle estimation. This process is similar to that of the (non-interferometric) radar altimeter but is more accurate because it utilizes RCMC processing and zero Doppler filtering.

Figure 18 shows the altimetry error for each scan plotted against the flight time axis, and Figure 19 shows the result as a histogram. We calculate the distance between the 3D coordinates of the red dots labelled as targets and the 3D coordinates of the light green lines representing the DEM in Figure 17 and take the smallest of these values as the altimetry error. The mean of the error is 13.55 m, and the standard deviation of the error is 27.87 m. Representing this as a Gaussian distribution results in the red graph shown in Figure 19. The Gaussian distribution is obtained using the least-squares approximation of the histogram.

**Figure 20:**
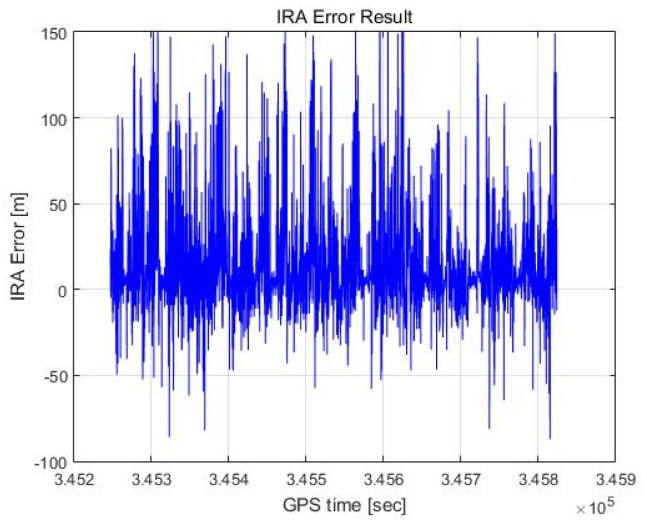
IRA error result (max point).

**Figure 21:**
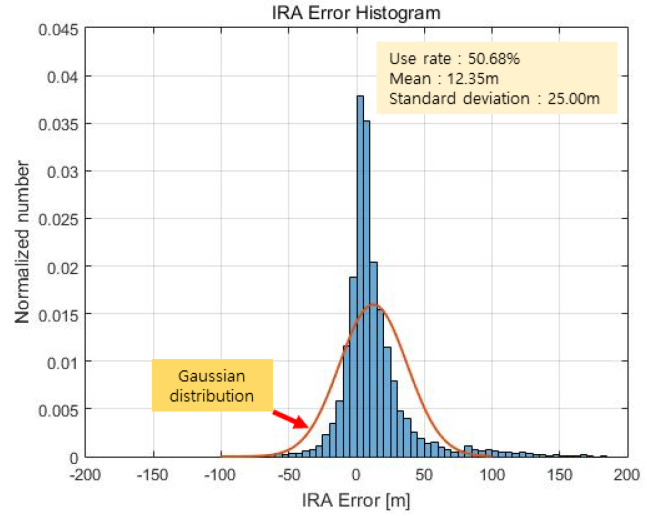
IRA error histogram (max point).

Figure 20 and Figure 21 show the altimetry results of the IRA based on the azimuth estimation of the maximum signal point. Considering the 3 dB beam width of the antenna, it is assumed that the estimated angle is valid if it is within $\theta_{width}$ (see Equation (14)). As a result, the valid rate of the angle is 50.68%, the mean of the error is 12.35 m, and the standard deviation of the error is 25.00 m. Representing this as a Gaussian distribution results in the red graph in Figure 21.

As shown in Figure 21, the histogram is significantly different from the Gaussian distribution because, as previously discussed in Section 2.6, the peak could be the signal reflected from a single target with a large RCS but could also be a superposition of signals from multiple targets with similar ranges and different angles. In other words, the deviation between a signal reflected from a single target and the superposition of signals from multiple targets is large such that the histogram appears to differ considerably from the calculated Gaussian distribution.

**Figure 22:**
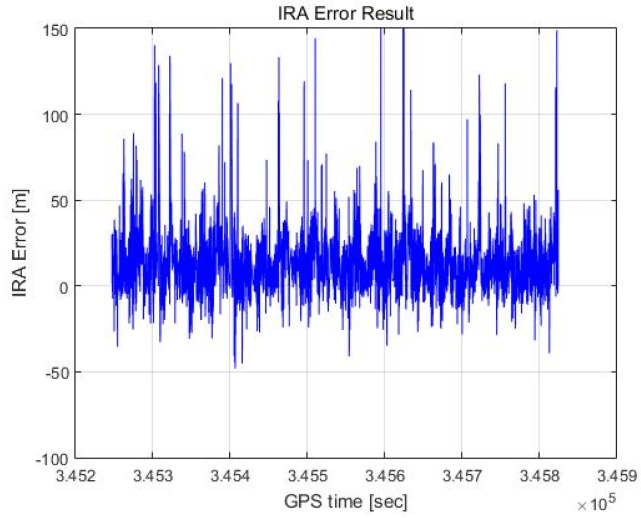
IRA error result (proposed method).

**Figure 23:**
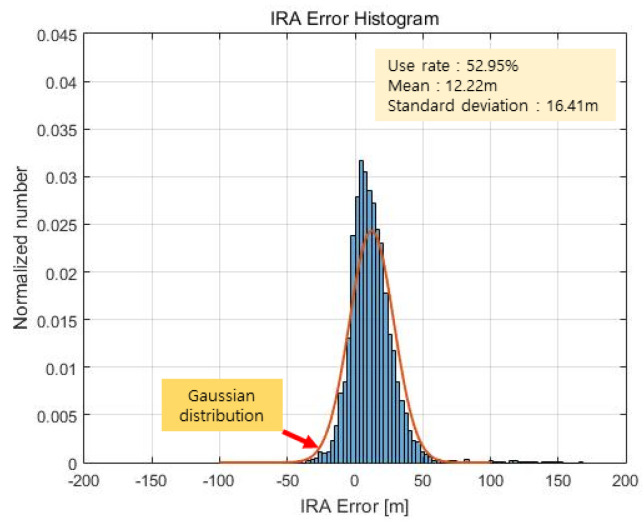
IRA error histogram (proposed method).

The altimetry results of the proposed method used to estimate the azimuth by evaluating the phase quality are shown in Figure 22 and Figure 23. Here, the angle valid rate is 52.95%, the mean of the error is 12.22 m, and the standard deviation of the error is calculated to be 16.41 m. This is represented by a Gaussian distribution, as represented by the red graph in Figure 23.

As shown in Figure 23, the histogram based on the proposed method appears to be similar to a Gaussian distribution, unlike that in Figure 21, and Figure 22 shows fewer extreme outliers and a smaller standard deviation of the error, unlike the outcomes in Figure 20. Table 1 summarizes the results for the three cases above.

In Table 1, the proposed method improves the standard deviation of the IRA. The circular error probability (CEP) of TRN depends on the accuracy of IRA. Thus, the smaller the standard deviation of the IRA, the more accurate the CEP of TRN. On the other hand, the mean values for the proposed and conventional methods are similar. There could be many reasons for this result. One of the reasons is that there may be some offset due to seasonal vegetation when the DEM is constructed and when the IRA measures the terrain.

The proposed method has the advantage of reducing the standard deviation but has the disadvantage of increasing the size of data due to the complexity of the processing procedure compared to the conventional method. However, the added procedures are all after the azimuth FFT, so the volume of data does not increase significantly.

## 4. Conclusions

In this paper, we proposed an altimetry method for IRA by evaluating the phase quality. Based on the *SNR* analysis between the terrain and IRA on the cruising aircraft, the LFM pulse is designed to obtain high-resolution range information, the RCMC processing in the time domain is designed based on the zero Doppler filtering of the DDA to obtain the elevation information of the target in the direct downward direction, and the PCM technique based on the conventional multiple baseline interferometry is employed to obtain the azimuth information using the phase difference of the received signal.

Then, the azimuths of a group of targets are computed by comparing the quality of the obtained phases. Finally, the azimuth information is estimated by selecting the most reliable target by evaluating their phase qualities. The test results verified that the proposed method can accurately calculate the altitude of the IRA. In the analysis process of the test results, it was found that the proposed method exhibits smaller standard deviation characteristics compared to the conventional method.

## Figures and Tables

**Table 1:** Comparison of test results by the algorithms.

Methods	Angle Valid Rate (%)	Mean (m)	Std (m)
RA (max point)	-	13.55	27.87
IRA (max point)	50.68	12.35	25.00
IRA (proposed)	52.95	12.22	16.41
